# Semantic representation and comparative analysis of physical activity sensor observations using MOX2-5 sensor in real and synthetic datasets: a proof-of-concept-study

**DOI:** 10.1038/s41598-024-55183-6

**Published:** 2024-02-26

**Authors:** Ayan Chatterjee, Martin W. Gerdes, Andreas Prinz, Michael A. Riegler, Santiago G. Martinez

**Affiliations:** 1 Department of Holistic Systems, Simula Metropolitan Center for Digital Engineering, Oslo, Norway; 2 Department of Information and Communication Technologies (ICT), Centre for E-Health, University of Agder, Grimstad, Norway; 3 Department of Health and Nursing Science, Centre for E-Health, University of Agder, Grimstad, Norway

**Keywords:** Semantic ontology, Semantic sensor network, General adversarial network, Gaussian Capula, MOX2-5, Multilayer perceptron, Synthetic data in healthcare, Health services, Public health

## Abstract

The widespread use of devices like mobile phones and wearables allows for automatic monitoring of human daily activities, generating vast datasets that offer insights into long-term human behavior. A structured and controlled data collection process is essential to unlock the full potential of this information. While wearable sensors for physical activity monitoring have gained significant traction in healthcare, sports science, and fitness applications, securing diverse and comprehensive datasets for research and algorithm development poses a notable challenge. In this proof-of-concept study, we underscore the significance of semantic representation in enhancing data interoperability and facilitating advanced analytics for physical activity sensor observations. Our approach focuses on enhancing the usability of physical activity datasets by employing a medical-grade (CE certified) sensor to generate synthetic datasets. Additionally, we provide insights into ethical considerations related to synthetic datasets. The study conducts a comparative analysis between real and synthetic activity datasets, assessing their effectiveness in mitigating model bias and promoting fairness in predictive analysis. We have created an ontology for semantically representing observations from physical activity sensors and conducted predictive analysis on data collected using MOX2-5 activity sensors. *Until now, there has been a lack of publicly available datasets for physical activity collected with MOX2-5 activity monitoring medical grade (CE certified) device.* The MOX2-5 captures and transmits high-resolution data, including activity intensity, weight-bearing, sedentary, standing, low, moderate, and vigorous physical activity, as well as steps per minute. Our dataset consists of physical activity data collected from 16 adults (Male: 12; Female: 4) over a period of 30–45 days (approximately 1.5 months), yielding a relatively small volume of 539 records. To address this limitation, we employ various synthetic data generation methods, such as Gaussian Capula (GC), Conditional Tabular General Adversarial Network (CTGAN), and Tabular General Adversarial Network (TABGAN), to augment the dataset with synthetic data. For both the authentic and synthetic datasets, we have developed a Multilayer Perceptron (MLP) classification model for accurately classifying daily physical activity levels. The findings underscore the effectiveness of semantic ontology in semantic search, knowledge representation, data integration, reasoning, and capturing meaningful relationships between data. The analysis supports the hypothesis that the efficiency of predictive models improves as the volume of additional synthetic training data increases. Ontology and Generative AI hold the potential to expedite advancements in behavioral monitoring research. The data presented, encompassing both real MOX2-5 and its synthetic counterpart, serves as a valuable resource for developing robust methods in activity type classification. Furthermore, it opens avenues for exploration into research directions related to synthetic data, including model efficiency, detection of generated data, and considerations regarding data privacy.

## Introduction

This section covers overview, motivation, novelty, and aim of the study.

## Overview

Regular physical activity is one of the most important contributors to our health. Physical activity improves brain health, manages weight, reduces chronic disease risk (e.g., diabetes type II, metabolic syndrome, cardiovascular disease, cholesterol level, blood pressure, and some cancers), strengthens bones and muscles, lowers symptoms of mental health (e.g., depression, anxiety), and improves individual ability to perform everyday activities, irrespective of age, abilities, and ethnicity^1–3^. The World Health Organization (WHO) defines physical activity as any body movement that requires energy-consuming skeletal muscles. Physical activities, including recreational sports, conveyance to and from places (movements), or as part of an individual's work. Both moderate and vigorous physical activity can improve health. Popular ways to be active include walking, cycling, running, weight exercise, and active recreation, and it can be practiced at any intensity level or age^3^. People who do not exercise enough have a 20–30% increased risk of death compared to those who are adequately active^3^. More than 80% of young people worldwide are not physically active enough^3^. WHO recommends that adults aged 18–64 should do at least 150–300 min (about 5 h) of moderate physical activity (MPA), or at least 75–150 min (about 2 and a half hours) of vigorous physical activity (VPA), or an equivalent combination of MPA and VPA throughout the week^3^. One possible way to prevent a decrease in physical activity and an increase in sedentary behavior is to use physical activity monitoring technology^4^. Monitoring daily physical activity towards the management of a healthy lifestyle goal has been a challenging task and one of the most prevalent research challenges in health informatics. However, this has been associated with more physical activity and less sedentary behavior^4^. Different smart devices (e.g., Fitbit, Garmin, Smartwatches, Sensewear Mini Armband, My Wellness Key Accelerometer, Actigraph, Pedometer, smartphone with installed applications) are available in the market to monitor and track fitness-related metrics (e.g., steps, VPA, MPA, low physical activity (LPA), sedentary bouts, calorie burnt, distance covered via running or walking) and related vital health signs (e.g., heart rate variability, respiratory rate, heart rate). The collected activity data is often available preprocessed (e.g., PMData^5^, Zenodo activity data^6^) or raw (e.g., UCI-HAR, WISDM, SHL, MD, HARTH, and AlgoSnap)^7^. Such data is seen as very important in the scientific research community. Several researchers have explored the use of sensors available in mobile devices to identify stationary activities for further applications in different scenarios related to ambient assisted living (AAL) and augmented living environments (ALE)^7^. Prior to this point, there has been a scarcity of openly accessible datasets capturing physical activity data using the MOX2-5 activity monitoring medical-grade (CE certified) device.

## Motivation

According to the scientific database searches, many articles reported their experiments on activity datasets collected by different wearable activity devices; however, most of the datasets are private; therefore, results are difficult to replicate or extend. Furthermore, the availability of high-quality, diverse, and sufficiently large datasets for training and evaluating algorithms remains a bottleneck in research and development. To address this challenge, we present a proof-of-concept study that utilizes the MOX2-5 activity sensor^8^ to generate a comprehensive dataset for physical activity monitoring. Synthetic datasets offer a promising solution to the problem of scarcity of real-world data, giving researchers and practitioners access to a wider range of scenarios and activities. This study not only releases the MOX2-5 dataset to the public but also showcases the viability and efficacy of synthetic datasets in enhancing the accessibility of training data for activity recognition models. The MOX2-5 dataset featured in this article offers preprocessed daily physical activity data.

One of the key aspects of our approach is the emphasis on semantic representation. We recognize the need for semantic enrichment of data to exploit the full potential of activity sensor observations. This semantic representation enables data interoperability, knowledge sharing, and advanced analytics. The Semantic Sensor Network (SSN) ontology represents sensor-related information (such as data repositories, processing services, and metadata) and observations and is therefore valuable in environments where sensor data and observations play an important role. SSN leverages Semantic Web technologies and ontologies to provide a standardized and machine-understandable way to describe, discover, and reason about sensors and sensor data. SSN is an important component of the Internet of Things (IoT) and the broader Semantic Web concept. They enable more intelligent, contextual, and data-driven applications by improving the understanding, discovery, and use of sensor data in various fields. Integrating real-world ontologies with SSNs can be more complex and requires careful modeling and adjustment to domain-specific standards and requirements. Our study includes a comprehensive comparative analysis between real and synthetic datasets. We evaluate the performance of activity recognition models trained on both data types, considering factors such as accuracy, robustness, and generalizability. Results reveal the utility of synthetic datasets and their potential to accelerate research progress and algorithm development in the field of physical activity monitoring.

## Novel contribution

This is an extended version of our previous study^9^. In this study, we have extended the semantic ontology design for annotating the sensor observations (e.g., our MOX2-5 physical activity datasets) with well-established SSN-based semantic information, elaborate the data collection process, and make the dataset public with its synthetic version. SSN is intended to promote the semantic interoperability between sensors and data systems. It standardized the way they describe and comprehend sensor data, this facilitates the communication and sharing of information between different systems and applications. A universal ontology that is common to all sensors may not have the same degree of standardized data output. In large-scale sensor networks and IoT applications, SSN can offer a scalable infrastructure for the management and comprehension of sensor data. Using a flexible SSN ontology-based knowledge-graph design solution, we lay the foundation for cross-IoT-domain collaboration and innovative research.

Use case and baseline we used the MOX2-5 dataset for daily activity-level classification with an MLP model as derived from our previous study^9^ where we compared the performance of the proposed MLP model with other state-of-the-art classifiers (such as Rocket, MiniRocket, MiniRocketVoting), and the proposed MLP model performed the best. Here, we explore how synthetic data enhanced training data to increase the performance of the used MLP model. We have shown a direction to predict daily physical activity levels into the following activity classes: sedentary (0), low (1), active (2), highly active (3), and vigorously active (4) with the MLP classification model. This proof-of-concept study addresses the generation of synthetic datasets based on the baseline MOX2-5 dataset and the semantic annotation of physical activity sensor observations with an SSN integrated OWL (Web Ontology Language) ontology. To verify the structural consistency, we use an ontology reasoner available in Protégé. We use SPARQL Protocol and RDF Query Language (SPARQL) for precise and efficient data retrieval and manipulation as a part of ontology verification^10,11^. We anticipate that our findings will contribute to the broader discussion on the role of synthetic data in data-scarce domains and the importance of semantic enrichment for meaningful and interoperable data. Additionally, we aim to provide insights into the practical applications of original and synthetic datasets in real-world scenarios, particularly in healthcare, fitness, and sports science. *According to the literature search, no similar studies have been found. Therefore, the contribution is novel*. Furthermore, we make the real and synthetic MOX2-5 datasets public in GitHub to practice open-access research with MOX2-5 dataset as a first study.

## Aim of the study

In healthcare, finding high-volume lifelogging data is challenging, and due to privacy and ethical issues, most datasets are private. Synthetic data generation techniques, such as GC^12^, CTGAN^13^, and TABGAN^14–16^, have been used for synthetic data generation with a focus on large-scale data sharing, experimentation, and analysis without revealing sensitive information. We have performed a comparative study with statistical metrics to find the best synthetic data generation method from our real MOX2-5 dataset. Moreover, we generate synthetic data from the best performing data generation method and contribute for open access. The MOX2-5 activity dataset and its synthetic version can be beneficial for other researchers for sedentary pattern analysis, posture detection and step forecasting. *Till date, not publicly available MOX2-5 activity datasets exist*. Thus, the main contributions of this work are summarized as follows.

1. We design and develop an ontology for semantification of observable and measurable physical activity sensor data and predictive analysis on the data.
2. We provide and open dataset containing MOX2-5 activity measurements and provide a baseline analysis of the data.
3. We provide synthetic data too, generated from the real values of the MOX2-5 dataset, and describe empirically the advantages of synthetic data generation in healthcare using well-established generative methods.
4. We evaluate the quality and the usefulness of the synthetic data.
5. We capture the risks and challenges in participant recruitment for sensor-based activity data collection.

## Methods

**Figure 1:**
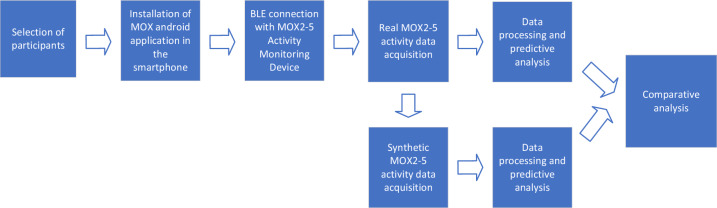
Workflow of the real and synthetic dataset creation and comparative analysis.

This section describes how we conducted our research, including data collection, ontology development, dataset generation, and analysis methods. Figure 1 represents the structure of the study for the data acquisition, processing, synthetic data generation and comparative analysis. This study used the Standards for Reporting Implementation (StaRI) checklist (see Supplementary Material-1). Data collection has been carried out in accordance with relevant guidelines and regulations in the “Ethics approval and consent to participate” section under Declarations. We followed the rules of the *General Data Protection Regulation (GDPR)*.

### Data collection

#### Participants and related distributions

Initially, we recruited twenty-five participants (19 men and 6 women) aged between 18 and 64; however, nine participants dropped in the middle of data collection due to medical reasons (e.g., pregnancy), lack of self-motivation, and device incompatibility issues. Therefore, the final data acquisition was performed with sixteen volunteering healthy individuals (12 men and 4 women) from Grimstad, Norway, for a period of 30–45 days (about 1 and a half months). We targeted normal-weight and overweight adults (based on BMI standards). The demographic statistics of the final population have been described in Table 1.

**Table 1:** Demographic characteristics of participants (N = 16).

Attributes	N%	Mean (µ)	Std (σ)
Gender			
Female	18.75		
Male	81.25		
Body composition			
Height		173.5	± 8.13
Weight		77.0	± 16.42
BMI		25.38	± 3.97
Education			
Bachelors	25.00		
Master’s	16.66		
Above Master’s	58.30		
Age		35.375	± 6.98

Regarding the gender distribution of our initially recruited participants for sensor-based data collection on physical activity, it has been important to emphasize that our study's primary focus lies in understanding broad patterns of physical activity across a diverse age range (18–64) rather than specifically examining gender-specific trends. While the initial participant demographics may skew toward a higher number of men, it has also been crucial to recognize that recruitment dynamics, individual preferences, and availability often influence the composition of study samples. Subsequent efforts will be made to actively address the gender balance in future participant recruitment to ensure a more representative dataset. Importantly, the study's overarching objective remains the investigation of physical activity behaviors within the specified age range, and the inclusion of participants from various genders is vital to achieving a comprehensive understanding of these patterns.

While the initial distribution may not perfectly mirror the general population's educational demographics, our recruitment strategy prioritized diversity in age to capture a broad spectrum of physical activity behaviors. Additionally, studies in the field of physical activity have often faced challenges in achieving a perfectly balanced representation across all demographic variables. The observed distribution may reflect the characteristics of individuals who were readily available and willing to participate in the study. Recognizing the importance of inclusivity, we acknowledge the feedback and intend to refine our recruitment strategies in future studies to ensure a more representative sample across various demographic factors, including education level, to enhance the generalizability of our findings.

#### Device information, value type, and specification

Our used MOX2-5 version 5 (MOX2-5) collects accelerometer data and processes the data. The MOX2-5 provides the following services over BLE communication, and all the services have three types of unique user identifiers (UUID): base, service, and short.

- Device information
  - Manufacturer information
  - Model number
  - Serial number
  - Hardware revision
  - Firmware revision
  - Software revision
- Battery
  - Battery level
- Device control
  - Commands
  - Status response
- Measurements
  - Request activity data
  - Activity data

The Measurement service is a custom service that reports the calculated algorithm values of the device to a host. The host requests the measured data by sending the “Request Activity Data” command with the correct parameters. Following this request, the device will continue to write collected values to the host until all write the host acknowledges actions and there are no values left. The device will now send an activity data update with the requested interval. The generated values of MOX2-5 algorithm are described in Table 2.

**Table 2:** Value populated through algorithm in MOX2-5.

Algorithm values	Description	Type
Timestamp	The timestamp represents the start time of this activity data window	32-bit unsigned
Upload status	The status activity data upload: H: History data, more data available in the device L: Live data, this is the last available record at this moment	8-bit unsigned
IMA sum	The sum of the calculated IMA values in this window [counts]	32-bit unsigned
Weight bearing	Total time that weight bearing is detected in this window [s]	12-bit unsigned
Sedentary classification	Total time that classification sedentary is detected in this window [s]	12-bit unsigned
Standing classification	Total time that classification standing is detected in this window [s]	12-bit unsigned
Class-LPA	Total time that classification LPA is detected in this window [s]	12-bit unsigned
Class-MPA	Total time that classification MPA is detected in this window [s]	12-bit unsigned
Class-VPA	Total time that classification VPA is detected in this window [s]	12-bit unsigned
Steps sum	The sum of measured steps in this window [counts]	16-bit unsigned

#### Sampling rate

Physical activity data in MOX2-5 sensors were collected continuously, throughout the day with Bluetooth (BLE) short-range wireless technology standard at a fixed sampling rate, which is typically around 1 Hz (1 sample per second) and in the comma-separated-version (CSV) format. The data was typically sampled and recorded at very short intervals, often in real-time or near-real-time.

#### Amplitude of the acceleration signal and movement intensity

The relationship between the amplitude of the acceleration signal and movement intensity (IMA) is directly proportional: as the higher the amplitude of the acceleration signal, the higher the movement intensity (IMA), and the higher the value of the counts per second will be. In the context of activity monitoring, the acceleration signal reflects the rate of change in velocity of a device or body part, which correlates with the intensity of physical movement. The Inertial Movement Analysis (IMA) quantifies this movement intensity based on the amplitude of the acceleration signal. When the amplitude is higher, it indicates more vigorous and energetic movements, such as running or jumping, resulting in an elevated IMA value. To quantify and measure these movements over time, the concept of "counts per second" is introduced. As the amplitude increases, the device registers a higher count of acceleration events per second, further emphasizing the link between amplitude, movement intensity, and the counts per second metric. This relationship is fundamental in interpreting and analyzing data from accelerometers or sensors, providing valuable insights into the dynamic and kinetic aspects of physical activities. The correlation between IMA and energy expenditure can be expressed in metabolic (MET)) values and established as:

- LPA: between 1.5 and 3 METS.
- MPA: between 3 and 6 METS.
- VPA: 6.0 or more METS.

For an upper leg MOX2-5 activity device placement, the corresponding IMA thresholds can be represented as:

- 4.5 < LPA ≤ 11.9 cycles per seconds (cps).
- 11.9 < MPA ≤ 26.8 cps.
- VPA > 26.8 cps.

Based on the observation, the relation between sedentary time and activity (LPA/MPA/VPA) time can be written as:

- $$\sum {\left( {{\text{sedentary}},{\text{ active}},{\text{ weight}} - {\text{bearing}},{\text{ standing}}} \right)} = {6}0 {\text{s}}\left( {{\text{sec}}.} \right).$$

Based on the observation and data patterns, during sleeping, the sedentary minutes goes as high as (≈ 58–60 s.) with IMA ≈ 0–20, step count ≈ 0, and activity time = 0.

#### Device wear location

**Figure 2:**
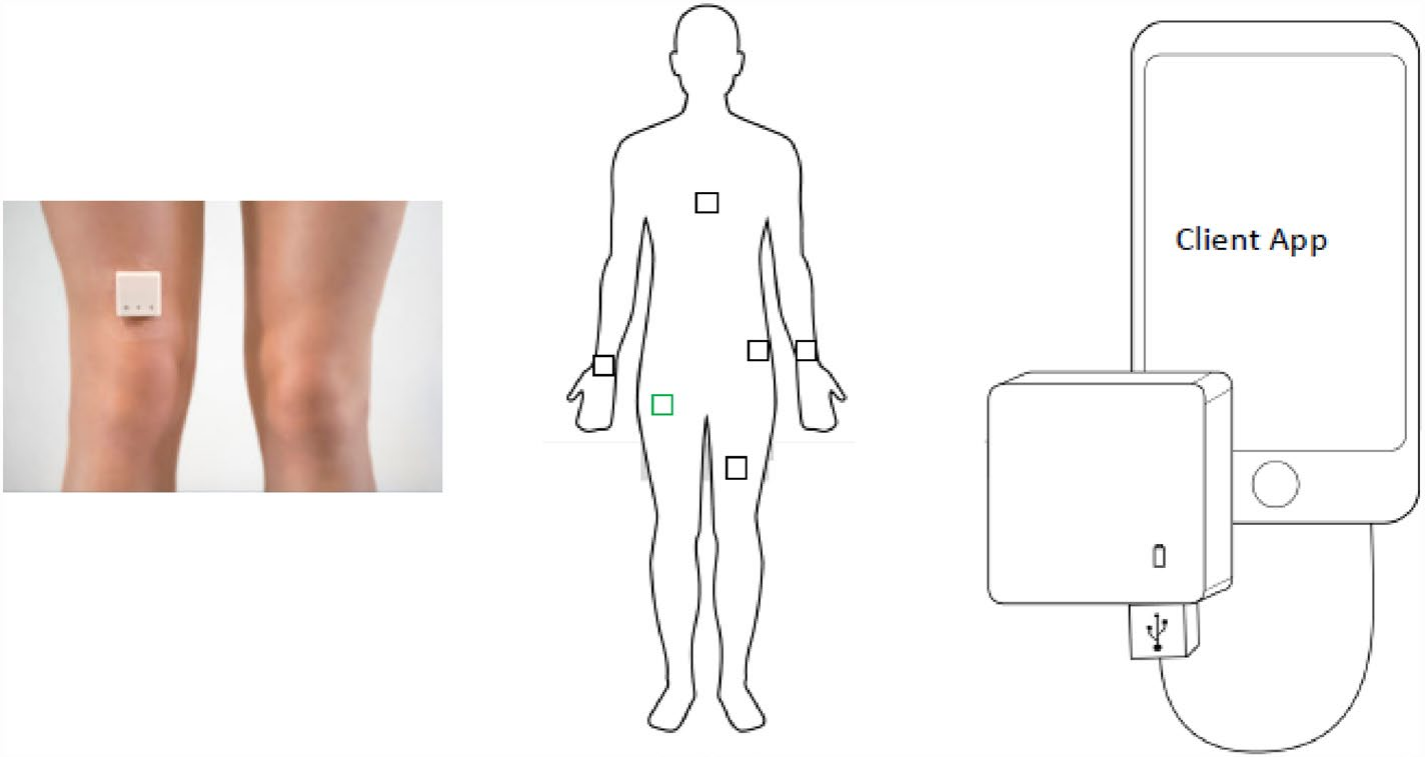
The wearing location of MOX2-5 activity sensor for data collection^8^.

The MOX2-5 activity sensor should be worn and placed in accordance with specific guidelines to ensure accurate data collection. The recommended placement typically involves securing the sensor to a specific part of the body as specified in Ref.^8^ (see Fig. 2), such as the thigh, hip, waist, and wrist, depending on the device design and the type of physical activity being monitored. It can also be worn on the chest; however, that is a separate version. Additionally, users were advised to wear the MOX2-5 sensor consistently during the designated period of data collection to maintain the integrity and reliability of the gathered information. Proper adherence to the specified wearing and placement instructions had been essential to obtain exact and significant insights into the individual's physical activity patterns.

We provided each participant a MOX2-5 activity device and supporting MOX android application to collect and store daily activity logging data in their android smartphone in the comma-separated value (CSV) format. The device captured required activity data for physical activity classification (e.g., physical activity intensity or IMA, LPA, MPA, VPA), daily step forecasting, and posture detection (e.g., sedentary (lying or sitting), standing, weight bearing, step count). With low power consumption, the MOX2-5 BLE device seamlessly measures and transfers high-resolution activity parameters. The used MOX2-5 activity monitor has the following specifications: dimensions as 35*35*10 mm, ultra-lightweight as 11 g, dust, and waterproof as IPX8, and durability of 2 years. The device has a battery life of 7 days or 60 days (about 2 months), with a Lithium Ion125 mAh rechargeable battery. The datasets presented in this paper include accelerometer sensor with parameters: sensitivity as 4 mg/LSB, sample rate as 25–100 H, and dynamic range of ± 8G.

#### Selection of activity sensor for this study case

Regarding the choice of MOX2-5 medical grade sensor for physical activity data collection, it has been essential to underscore the distinctive advantages offered by these sensors in the context of this study. MOX2-5 sensors provide medical-grade precision in capturing physiological parameters during physical activity, enabling a nuanced analysis of participants' responses. While alternative sensors such as accelerometers, video cameras, and gas chemical sensors are indeed valuable in specific applications, the MOX2-5 sensors specifically excel in offering real-time, high-fidelity data on physical activity changes. This level of granularity is crucial for understanding the intricacies of physiological responses during diverse physical activities. The selection of MOX2-5 sensors aligns with the precise objectives of our research, allowing us to contribute in-depth insights into the physiological aspects of physical activity and the potential applications of medical-grade sensor technology in the health monitoring domain.

#### Data acquisition procedure

MOX2-5 activity monitoring devices are manufactured by Maastricht Instruments, a spin-off company of the Maastricht Hospital. They provided us with activity monitoring devices equipped with chargers and a MOX android mobile application. The MOX2-5 device is portable, has a unique MAC address, and a small internal storage for collecting activity data for a week. Before the MOX can be used, the mobile application must be installed on an android compatible smartphone and connected to the MOX2-5 activity device over BLE. After that, the MOX2-5 device can be placed on the preferred wearing location. The MOX2-5 device continuously monitors physical activities based on the accelerometer data and initially stores collected data in its internal storage and followed by, based on connection establishment with the mobile application, transfers activity data to the smartphone for persistence in the CSV format. The accelerometer sensor is a tri-axial sensor with the co-ordinate variables X, Y, and Z. During the measurement, BLE connection and data must be checked continuously. The MOX2-5 device must be disconnected, removed from the wearing location, and charged when the LED will turn into “ORANGE”. The “Download” folder of the smartphone stores daily activity files in CSV format that holds activity records per minute. The provided MOX app by Maastricht Instruments is not compatible with android version > 9.0 and < 7.0. Therefore, it created version compatibility issues in certain participants.

### Proposed ontology model for semantic representation

An ontology is a formal and unambiguous representation of knowledge or information about a specific domain of interest. It serves as a structured, common vocabulary or framework for describing concepts, entities, their properties, and their relationships within the domain.

#### Ontology vs. databases

Ontologies are suitable for knowledge representation, semantic search, data integration, reasoning, and applications where capturing the meaning and relationships between data entities is critical. They are commonly used in areas such as the Semantic Web, healthcare (for medical ontologies), and scientific research. The ontology knowledge graph can grow based on the open-world assumptions. In contrast, databases are ideal for applications that require efficient data storage, retrieval, and transaction management. They are widely used in business applications, e-commerce, finance, customer relationship management (CRM), and many other areas that require structured data management.

#### Ontology structure

Representing an ontology using tuples is a simple and intuitive approach where it uses ordered sets of elements to describe the ontology's structure. Each tuple represents a fact or relationship within the ontology. The Mathematical representations of ontology involve formal logic and set theory. Ontologies describe concepts, relationships, and axioms that can be represented mathematically using symbolic notation. Some common mathematical representations and concepts used in ontology modeling are in Textbox 1. Ontologies written in Web Ontology Language (OWL) typically consist of several key components that define structures, classes, individuals, properties (data and object), axioms, restrictions, annotations, logical axioms, and namespaces. These components help formalize knowledge in a machine-readable format. OWL ontologies can become more complex by adding multiple classes, properties, axioms, and imports, allowing formal representation and automated reasoning of complex knowledge structures.

**Textbox 1:** The mathematical representations and concepts used in ontology modeling.

1. Description Logic: It uses mathematical notations to represent concepts (classes), individuals, and relationships (properties) 2. First-Order Logic: It involves quantifiers (∀ for "for all" and ∃ for "there exists") and logical operators (¬ for "not," ∧ for "and," ∨ for "or," → for "implies") 3. Set Theory: It uses notation like ∪ (union), ∩ (intersection), and ⊆ (subset) to represent relationships between sets of individuals or concepts 4. Predicate Calculus: It involves predicates (relations) and variables 5. Graph Theory: It uses mathematical notation to represent nodes (concepts or individuals) and edges (relationships) in the ontology graph 6. Axiomatic Set Theory: It involves a set of axioms that define set theory mathematically, and ontological concepts can be mapped to sets	

#### Proposed ontology

Creating a complete ontology for observing physical activity sensors, integrating it with the SSN ontology, and deploying SPARQL queries to query the integrated ontology is a complex task that requires careful design and extensive development. In our designed and developed ontology model, we have integrated the concepts with SSN ontology and for the same, we align the classes and properties in our ontology with SSN's classes and properties. We use “Observation” class to represent our “PhysicalActivityObservation” and properties like “observedBySensor” with SSN's properties for sensor observations. Our ontology model consists of the following elements as described in Textbox 2.

**Textbox 2:** The elements in our ontology.

Classes PhysicalActivityObservation: Represents an observation of physical activity ActivityLevel: Represents different activity levels (e.g., sedentary, light, moderate, vigorous) based on the predictive analysis SedentaryTimeObservation: Represents an observation of sedentary time StepsObservation: Represents an observation of the number of steps taken Sensor: Represents the sensors used for observation Object properties observedActivityLevel: Relates a PhysicalActivityObservation to an ActivityLevel observedSteps: Relates a PhysicalActivityObservation to a StepsObservation observedSedentaryTime: Relates a PhysicalActivityObservation to a SedentaryTimeObservation observedBySensor: Relates an observation to the Sensor Data properties observationTime: Represents the time of observation predictedActivityLevel: Represents the predicted activity level for a person hasPerson: Relates an observation to a specific person	

The proposed ontology supports personalization, and the OWL representation of the same concept has been captured in Supplementary Material-2 for an individual. For the verification of our ontology model, we use MOX2-5 activity sensor’s observation data. However, the proposed ontology can be aligned with other wearable sensors for behavioral monitoring.

Analyzing the complexity of the proposed ontology involves evaluating various aspects of the ontology's structure, content, and reasoning requirements. We achieve the same with the following considerations—measuring the size of the ontology in terms of the number of classes, individuals, properties, and axioms, analyzing the depth of the class hierarchy, assessing the number of object properties and data properties in the ontology, evaluating the number of axioms in the ontology, including subclass axioms, equivalence axioms, disjoint axioms, and property restrictions, analyzing the use of cardinality constraints (e.g., min, max, some) on properties, considering the use of complex data types and restrictions on data properties, determining the type of reasoning and inference required, identifying the OWL profile (e.g., OWL Full in this case), assessing consistency, modular design patterns, and SPARQL query complexity.

#### Activity level classification with MLP model

The relevant features obtained from the MOX2-5 activity device are—timestamp, IMA, sedentary seconds, weight-bearing seconds, standing seconds, LPA seconds, MPA seconds, VPA seconds, and steps per minute. The “step” and “IMA” are the most valuable and robust features of the MOX2-5 sensor-based datasets, as other attributes (except the timestamp) are derived from these (e.g., LPA, MPA, and VPA are derived from IMA as defined in Table 3). IMA has a strong relation with steps where steps are primarily involved as a measure for activities. In the MOX2-5 sensor, sedentary time refers to the non-activity duration, including leisure and sleep. Therefore, one cannot see if it’s sleep or just not doing anything else.

**Table 3:** The relation between IMA and activity levels as per MOX2-5 algorithm.

Activity type	Rule
LPA	0 ≤ IMA ≤ 400
MPA	401 ≤ IMA ≤ 800
VPA	IMA ≥ 801

To determine feature importance, we used traditional methods, such as the SelectKBest univariate feature selection with Chi-squared test from the sklearn Python library^4,17^ and ExtraTreesClassifier^4,17^ to cross verify the selected features. The data is non-gaussian in nature; thus, we used spearman correlation^4,17^ analysis to explore the association between the features. We removed features with a high correlation coefficient (|r|) value. Moreover, we have used the forward and backward filling and averaging methods to handle missing data. We handled outliers with boxplot analysis. After handling missing data and outliers, we converted individual activity data from /minute entry to /day entry. On the resulting dataset, we applied standard rules defined in Table 4 to generate an activity level class for a multi-class classification problem. Captured time-series activity data are continuous in nature; however, we converted it into discrete tabular form for such classification problem after removing the “Timestamp” feature. Both the final and processed tabular data and its synthetic versions are part of the dataset.

**Table 4:** “Activity Level” class creation based on standard rules for multi-class classification problem.

Activity level	Rule*	Active (encoded)
Sedentary	((Steps < 5000) ∧ (VPA*2 + MPA)*7 < 90 ∧ LPA ≥ 0)) ˅ (Steps < 5000)	0
Low active	((Steps > 4999) ∧ (VPA*2 + MPA)*7 ≥ 90 ∧ (VPA*2 + MPA)*7 < 210) ˅ (Steps > 4999 ∧ Steps < 7500)	1
Active	((Steps > 4999) ∧ (VPA*2 + MPA)*7 ≥ 210 ∧ (VPA*2 + MPA)*7 < 300) ˅ (Steps > 7499 ∧ Steps < 10,000)	2
Medium active	((Steps > 4999) ∧ (VPA*2 + MPA)*7 ≥ 300 ∧ (VPA*2 + MPA)*7 < 360)) ˅ (Steps > 9999 ∧ Steps < 12,500)	3
Highly active	((Steps > 4999) ∧ (VPA*2 + MPA)*7 ≥ 360) ˅ (Steps > 12,499)	4

*MPA = 2VPA.

To classify real and its different combination with synthetic activity data, we designed and developed an MLP model which is inspired by the architecture of fully connected neural network (FCNN) with fivefold cross validation and the “ReduceLROnPlateau” method^17^. The designed and developed MLP model consisted of six layers (first five layers with ReLU activation function and the last layer with the SoftMax activation function). The ReLU function resolves vanishing-gradient problem and helps in efficient convergence. We used the categorical cross entropy loss function in our MLP model compilation as our dependent class had been one-hot encoded. We used ADAM optimizer as it is time and memory wise efficient. In Keras, the default ADAM configuration is α = 0.001, β_1_ = 0.9, β_2_ = 0.999, € = 1e − 08 and Decay = 0.0, and we used a similar configuration in this experiment. We captured the loss histories to compare training and test losses over multiple epochs. ADAM adjusts the learning rate of each parameter individually, allowing it to cope with various optimization challenges, such as vanishing or exploding gradients. ReLU replaces negative input values with zeros, which adds nonlinearity to the model while enabling faster training and better convergence. Its piecewise linear behavior promotes sparsity in neural activations and enables the network to efficiently learn complex patterns and representations.

### Synthetic tabular dataset generation

Creating synthetic data is becoming increasingly important due to privacy concerns and data availability. Synthetic data can help to anonymize individuals while preserving the distributional nature of the data which could allow for easier sharing. Further, synthetic data can help to increase the number of samples in a dataset and increase the performance of models. We used the following methods with the tabular real MOX2-5 activity data to generate synthetic data efficiently.

#### Gaussian Capula (GC)

The Gaussian copula is a statistical modelling technique for data synthesis. Copula allows us to decompose a joint probability distribution into marginal values of uncorrelated variables and functions that "couple" these marginal values together. Copulas are multivariate distributions with embedded relevant information. Gaussian copulas are multivariate normal distributions with learned dependencies. The high-level steps for synthetic data generation with GC method have been detailed in Textbox 3. We used python SDV package^18^ and “GaussianMultivariate” method to generate synthetic data with GC.

**Textbox 3:** Steps for synthetic data generation with GC method.

1. Know the probability distribution for each column in the table 2. Use the inverse CDF transformation of the standard normal to them (i.e., convert the distribution of the column to a normal distribution) 3. Learn about the correlations of these newly generated random variables to create a copula model, and 4. Samples from a multivariate standard normal distribution with learned correlation	

#### CTGAN

Conditional Tabular Generative Adversarial Networks (CTGAN) is a deep learning data synthesis technique. As the name suggests, this is a GAN-based approach. A standard GAN architecture consists of two neural networks: one acts as a generator, which takes some input and generates synthetic data. Then have a second neural network that serves as a discriminator to see if they can distinguish actual data from synthetic data. The results of the discriminator are fed back to the generator to help the generator produce better synthetic output. The CTGAN architecture introduces a conditional generator that generates rows conditioned on one of the discrete columns and the training data based on the protocol samples, instead of feeding the generator with random training data that may not adequately represent subcategories of highly imbalanced categorical columns—the frequency of each column category for this discrete column. This helps the GAN model to explore all possible discrete values uniformly (not necessarily uniformly). The CTGAN represents continuous columns with mode-specific normalization. We used the python SVD package and “CTGANSynthesizer” module to generate synthetic tabular data with CTGAN.

#### TABGAN

Currently, GANs are widely used to generate image data; however, they can be used to create synthetic tabular data from scratch. GANs can generate synthetic data from scratch and consist of two parts: a generator and a discriminator. The generator is used to generate synthetic data from random noise in the input; the discriminator is used to classify whether a sample is real or synthetic (as generated by the generator). The power of the discriminator is used to update and optimize the generator and discriminator. We followed the steps as described in Textbox 4 to design and develop a TABGAN in Keras with TensorFlow as backend.

**Textbox 4:** Steps for synthetic data generation with TABGAN method.

1. We created random noise in the latent space and reshaped it to the dimensions for matching the input of generator model using the generate_latent_points method 2. To produce “n” synthetic samples with class labels, we defined the generate_fake_samples method 3. We created input for the generator from latent points or random noise 4. The Generator model generated “n” samples based on predicting input random noise and label the real data with “1” and synthetic data with “0” 5. We created the discriminator model 6. We made weights in the discriminator not trainable and defined the GAN model with two input models—Generator and Discriminator 7. We trained the GAN model with generator, discriminator, GAN model, and latent dimension, and saved the based model for further use	

Our sequential Generator model had three Dense layers with two layers activated by the “ReLU” activation function. The output layer was activated by the “linear” function. We initialized the Kernel by “he_uniform”. We maintained the dimensions of the output layer like the dimensions of the dataset. The discriminator model consisted of three Dense layers. The first two layers were activated by the “ReLU” activation function, and the output layer was activated by the “Sigmoid” activation function to discriminate the real (True or 1) and synthetic (False or 0) data. We compiled the Discriminator model with optimizer as “ADAM” and loss function as “binary_crossentropy”. Moreover, the combined Generator and Discriminator model was compiled with “ADAM” optimizer and “binary_crossentropy” loss function.

### Performance metrics

#### Classification

The performance of the designed and developed MLP classification model has been evaluated against precision, recall, specificity, accuracy score, F1 score, classification report, and confusion matrix^4,17,19,20^. A confusion matrix is a 2-D table (“actual” vs “predicted”), and both dimensions have “True Positives (TP)”, “False Positives (FP)”, “True Negatives (TN)”, and “False Negatives (FN)”. Equations to calculate classification metrices are

$$ {\text{Accuracy}}\left( {\text{A}} \right) = \frac{{\left( {{\text{TP}} + {\text{TN}}} \right)}}{{\left( {{\text{TP}} + {\text{FP}} + {\text{FN}} + {\text{TN}}} \right)}},0 \le \frac{{\left( {\text{A}} \right)~}}{{\left( {100} \right)}} \le 1 $$

$$ {\text{Precision}}\left( {\text{P}} \right) = \frac{{\left( {{\text{TP}}} \right)~}}{{\left( {{\text{TP}} + {\text{FP}}} \right)}} $$

$$ {\text{Recall}}\left( {\text{R}} \right){\text{or}}\;{\text{Sensitivity}}\;\left( {\text{S}} \right)\;{\text{or}}\;{\text{True}}\;{\text{positive}}\;{\text{rate}} = \frac{{\left( {{\text{TP}}} \right)~}}{{\left( {{\text{TP}} + {\text{FN}}} \right)}} $$

$$ {\text{Specificity}}\left( {\text{S}} \right) = \left( {1 - {\text{Sensitivity}}} \right) = \frac{{\left( {{\text{TN}}} \right)~}}{{\left( {{\text{TN}} + {\text{FP}}} \right)}} $$

$$ {\text{F}}1\;{\text{score}}\left( {{\text{F}}1} \right) = \frac{{\left( {2{\text{*P*R}}} \right)}}{{\left( {{\text{P}} + {\text{R}}} \right)}}, \le \frac{{\left( {{\text{F}}1} \right)~}}{{\left( {100} \right)}} \le 1 $$

$$ {\text{Matthew's}}\;{\text{correlation}}\;{\text{coefficient}}\;\left( {{\text{MCC}}} \right) = \frac{{\left( {{\text{TP}}\left( {{\text{TP*TN}}~ - ~{\text{FP*FN}}} \right)} \right)}}{{\sqrt {\left( {{\text{TP}} + {\text{FP}}} \right)\left( {{\text{TP}} + {\text{FN}}} \right)\left( {{\text{TN}} + {\text{FP}}} \right)\left( {{\text{TN}} + {\text{FN}}} \right)} }}, - 1 \le \frac{{\left( {{\text{MCC}}} \right)~}}{{\left( {100} \right)}}~ \le ~ + 1. $$

Accuracy tells how close a measured value is to the actual one. Recall or sensitivity suggests the exact number of positive measures. Precision means how relative the measured value is to the actual one.

#### Synthetic data quality evaluation

We used “Classifier F1-scores and their Jaccard similarities” to evaluate the quality of the generated synthetic data with “Table_evaluator” python library^21^. The Jaccard Similarity Score is a versatile and widely applicable metric that provides a simple and intuitive measure of similarity between sets. Here, it is used for comparing sets by measuring the similarity of their elements.

$$ {\text{J}}({\text{A}},{\text{B}}) = \left| {{\text{A}} \cap {\text{B}}} \right|/\left| {{\text{A}} \cup {\text{B}}} \right| $$

where, J = Jaccard distance, A = Set-1, B = Set-2, A and B are sets

$$ {\text{RMSE}} = \sqrt {\sum\limits_{{i = 1}}^{n} {\left( {Y_{i}  - \widehat{{Y_{i} }}} \right)^{2} /n} }  $$

where ${Y}_{i}$ = actual, $\widehat{{Y}_{i}}$ = predicted, n = total population~

$${\text{MAE}} = \mathop \sum \limits_{{i = 1}}^{n} {\raise0.7ex\hbox{${\left| {Y_{i}  - \widehat{{Y_{i} }}} \right|}$} \!\mathord{\left/ {\vphantom {{\left| {Y_{i}  - \widehat{{Y_{i} }}} \right|} n}}\right.\kern-\nulldelimiterspace} \!\lower0.7ex\hbox{$n$}}$$

where ${Y}_{i}$ = actual, $\widehat{{Y}_{i}}$ = predicted, n = total population.

In the equation, X and Y are data objects represented by vectors. The similarity value is the dot product of X and Y divided by the squared magnitude of X and Y minus the dot product. The average nearest neighbor is calculated as the observed average distance divided by the expected average distance^21^.

Moreover, we used the Ordinary Least Squares (OLS)^22^ using “Statsmodels” to compare the real and the synthetic datasets. The OLS used the following metrics for performance measurement:

$$ {\text{R}}^{{2}} = {1}{-}{\text{RSS}}/{\text{TSS}}, $$

where R^2^ = coefficient of determination, RSS = sum of squares of residuals, TSS = total sum of squares

$$ {\text{Adjusted R}}^{{2}} = {1}{-}\left( {\left( {{1}{-}{\text{R}}^{{2}} } \right)\left( {{\text{N}} - {1}} \right)} \right)/\left( {{\text{N}} - {\text{p}} - {1}} \right), $$

where R^2^ = sample R-squared, N = total sample size, p = number of independent variables

$${\text{Residual Standard Error }}\left( {{\text{RSE}}} \right) = \sqrt {\mathop \sum \limits_{{i = 1}}^{n} {\raise0.7ex\hbox{${\left( {Y_{i}  -  \widehat{{Y_{i} }}} \right)^{2} }$} \!\mathord{\left/ {\vphantom {{\left( {Y_{i}  -  \widehat{{Y_{i} }}} \right)^{2} } {df}}}\right.\kern-\nulldelimiterspace} \!\lower0.7ex\hbox{${df}$}}}$$

where ${Y}_{i}$ = actual, $\widehat{{Y}_{i}}$ = predicted, df = degree of freedom.

F-value = Larger sample variance/Smaller sample variance = $\frac{{S}_{1}^{2}}{{S}_{2}^{2}}$, Where S = standard deviation.

The main difference between Adjusted R-squared and R-squared is simple, adjusted value considers various independent variables and tests them against the model whereas R-squared does not. Adjusted R-squared is always less than or equal to R-squared. Larger R-squared means the model is better means the model is better. RSE is the standard deviation of the residuals. An F-test is any statistical test in which the test statistic has an F-distribution under the null hypothesis. It is most often used when comparing statistical models fitted to a dataset to identify the model that best fits the population from which the data were sampled. A comparative analysis helped us to identify the best synthetic tabular data generation method in this context for further data augmentation perspective. Furthermore, it helped to determine if the samples are coming from the same distribution or not.

### Ethical approval and consent to participate

We received approval from the Regional Committees for Medical and Health Research Ethics (REK) (#53224) to execute the project. We received ethical approval from the Norwegian Centre for Research Data (NSD) or Norwegian Agency for Shared Services in Education and Research (SIKT) (#797208). For the data collection, informed or signed consent has been obtained from all the participants. Overall, we used GDPR guidelines for personalized data security and privacy (data governance). Participants had the right to own and view their personal data without tampering.

## Results

This section consists of data records, experimental setup, and experimental results. The experimental results elaborate evaluation of the proposed ontology and predictive analysis.

### Data records

We collected physical activity data from 16 participants with MOX2-5 wearable activity device *(MOX2_5_data_unlabelled.csv and MOX2_5_data_labelled.csv in the *Supplementary Material-3*)*. The detailed description of the dataset is provided in Tables 5 and 6.

**Table 5:** MOX2-5 activity data details for participants (N = 16).

Participant(s)	Duration of data collection (days)	Considered Total records	Total sedentary seconds	Total VPA seconds	Total MPA seconds	Total LPA seconds	Total steps
P1	43	43	3,512,792	8510	52,196	183,702	392,512
P2	48	48	4,261,190	50,214	95,730	200,524	588,132
P3	30	30	2,293,208	24,248	62,502	65,494	273,708
P4	31	31	3,065,884	15,156	23,402	254,332	442,365
P5	30	30	2,402,790	43,104	57,606	123,170	398,029
P6	30	30	2,316,338	51,094	64,885	77,141	305,673
P7	39	39	3,784,340	78,908	53,876	245,160	398,296
P8	31	31	3,028,756	112	38,230	103,480	252,551
P9	32	32	2,623,966	30,722	72,308	153,174	419,063
P10	31	31	2,395,160	27,024	58,846	120,820	347,144
P11	33	33	3,061,236	15,432	45,440	247,896	436,404
P12	31	31	590,028	25,142	37,680	151,150	271,888
P13	31	31	2,297,915	10,006	27,487	135,314	269,258
P14	30	30	1,963,218	14,891	39,670	193,226	320,134
P15	38	38	925,614	256,896	58,212	32,272	411,033
P16	31	31	664,302	18,746	63,638	187,498	341,063

**Table 6:** Participant characteristics (n = 16).

Factors	Mean (µ)	SD (σ)	Min	Max
Age	35.375	7.03	21	51
Height (cm)	173.5	8.02	158.5	184.0
Weight (kg)	77.0	16.36	55.0	107.0
BMI	25.38	3.93	19.41	31.604
Duration	33.6875	5.41	30	48
Total sedentary minutes	2,449,171	1,051,610.5	590,028	4,261,190
Total VPA minutes	41,887.81	60,688.5	112	256,896
Total MPA minutes	53,231.75	17,965	23,402	95,730
Total LPA minutes	154,647.1	66,540.6	32,272	254,332
Total steps	366,703.3	87,202.25	252,551	588,132

**Figure 3:**
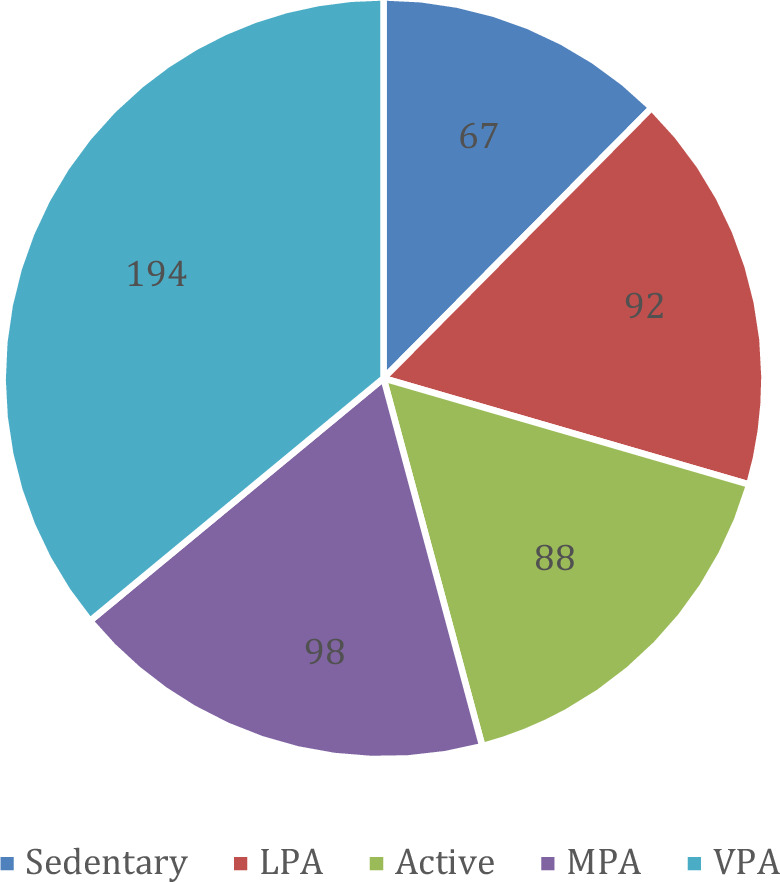
The class distribution for the MOX2-5 dataset in the pie-chart.

The total size of the datasets is 42 Kilobytes (KB) containing 539 unique measurements. Based on the feature ranking, we selected the best five features for predictive analysis—sedentary, LPA, MPA, VPA, and steps. The class distribution for the predictive analysis has been depicted in Fig. 3. We used a similar dataset for synthetic data generation with GC, CTGAN, and TBGAN. We termed the real data as R, GC populated synthetic data as FGC, CTGAN generated synthetic data as FC, and the TBGAN generated synthetic data as FT. It results in the following data in Supplementary Material-4* (*synthetic_data_GC_labelled.csv, synthetic_data_CTGAN_labelled.csv, and synthetic_data_TBGAN_labelled.csv) of total 88 KB in volume and they are used in this paper for experiments. The class distribution of FGC dataset, FC dataset, and FT dataset have been described in Tables 7, 8 and 9.

**Table 7:** Description of the FGC datasets.

	Count	Mean	Std	Min	25%	50%	75%	Max
Sedentary	539	75,539.8	33,222.5	− 12,580.6	57,511.8	77,359.9	92,221.6	186,288.3
LPA	539	4861.79	2951.31	322.38	2664.50	4399.28	6333.91	19,853.06
MPA	539	1520.64	1160.05	− 87.49	636.20	1193.40	2197.69	5804.55
VPA	539	730.65	1121.41	− 1028.60	33.30	495.21	1006.86	6921.35
Steps	539	10,588.66	5385.92	− 2376.91	6674.32	9865.35	13,758.37	32,111.69
Active	539	2.37	1.42	0.00	1.00	2.00	4.00	4.00

**Table 8:** Description of the FC datasets.

	Count	Mean	Std	Min	25%	50%	75%	Max
Sedentary	539	48,557.4	30,301.9	3172.0	20,627.5	59,056.5	69,996.5	156,329.0
LPA	539	7004.91	3820.56	126.0	4254.50	6317.0	8813.5	18,709.0
MPA	539	1496.56	1466.66	0.0	529.0	1084.0	2047.0	8038.0
VPA	539	885.49	1456.75	0.0	0.0	317.0	1075.5	9714.0
Steps	539	12,116.05	5956.09	345.0	6674.32	12,169.0	15,749.5	42,815.0
Active	539	2.76	1.43	0.00	2.00	3.00	4.00	4.00

**Table 9:** Description of the FT datasets.

	Count	Mean	Std	Min	25%	50%	75%	Max
Sedentary	539	8641.4	2127.3	4283.0	7166.7	8405.3	10,100.5	16,063.0
LPA	539	902.23	279.61	346.2	688.50	862.52	1081.63	1909.88
MPA	539	− 180.44	150.69	− 614.83	− 285.63	− 176.18	− 62.73	188.35
VPA	539	272.85	76.11	114.34	219.66	264.21	322.92	528.26
Steps	539	1836.82	425.53	852.06	1504.09	1793.63	2102.72	3397.36
Active	539	0.00	0.00	0.00	0.00	0.00	0.00	0.00

**Figure 4:**
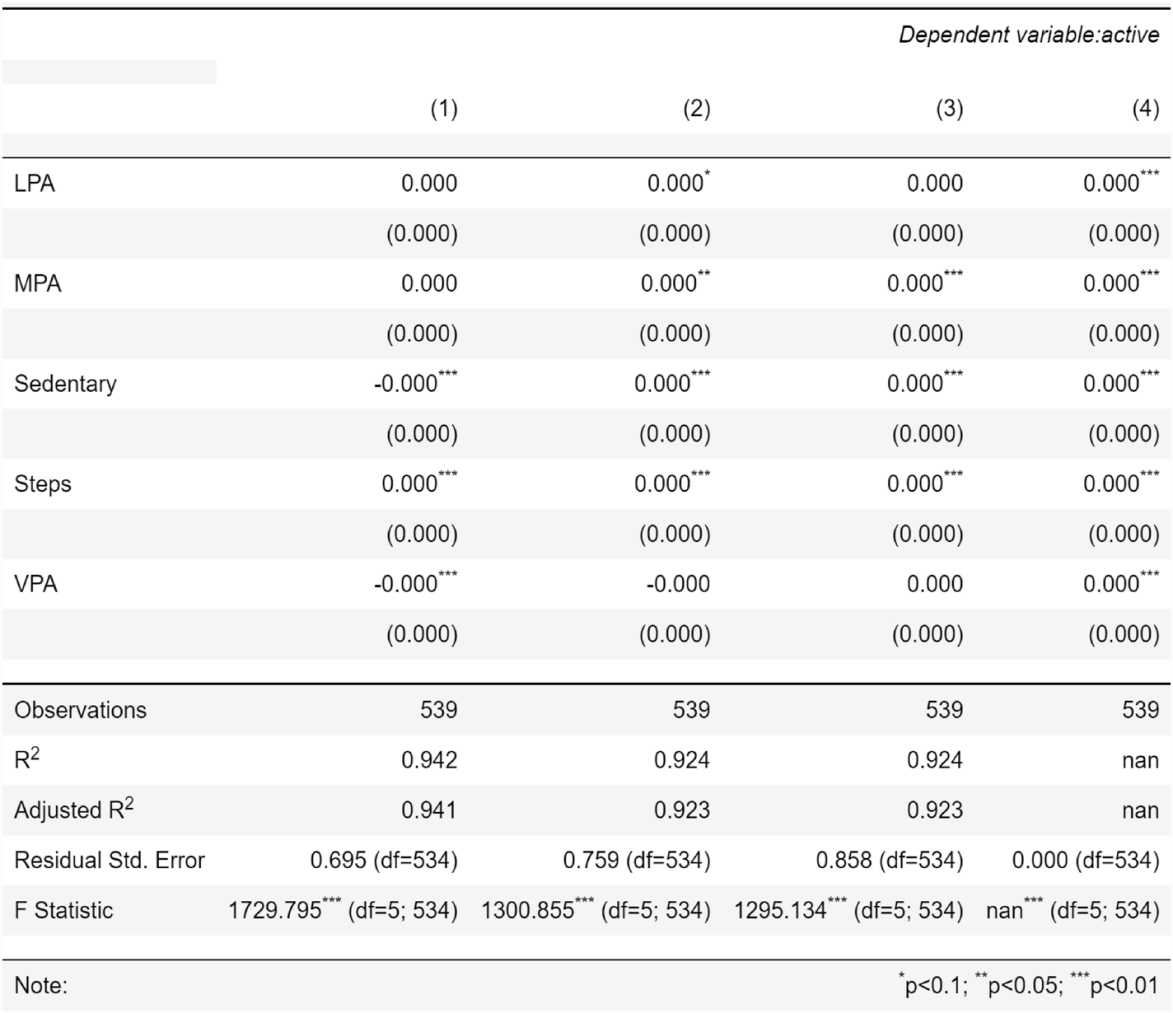
The comparison between R (1), FGC (2), FC (3), and FT (4) datasets.

**Figure 5:**
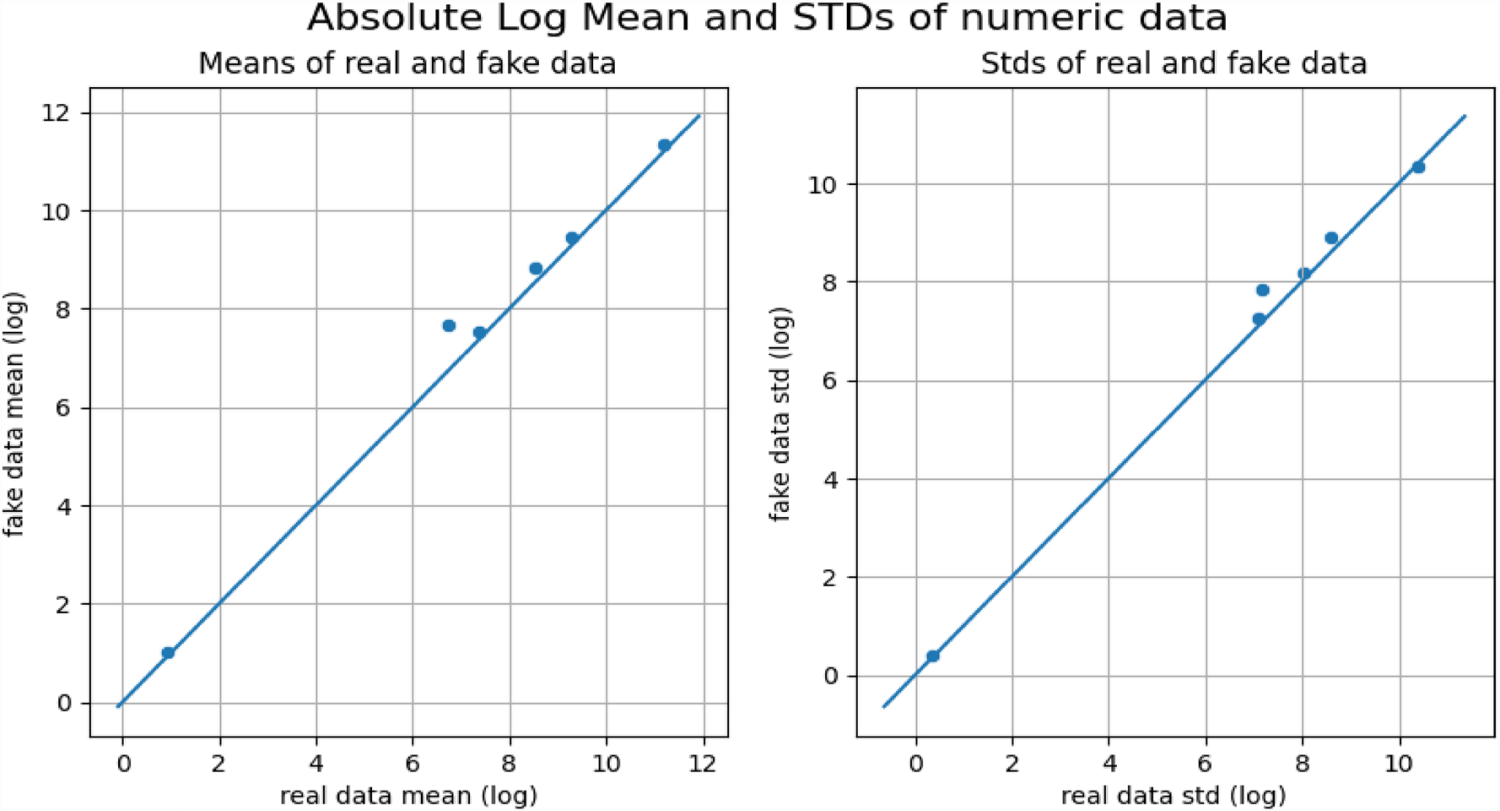
The absolute log means and std between the R and FC datasets.

**Figure 6:**
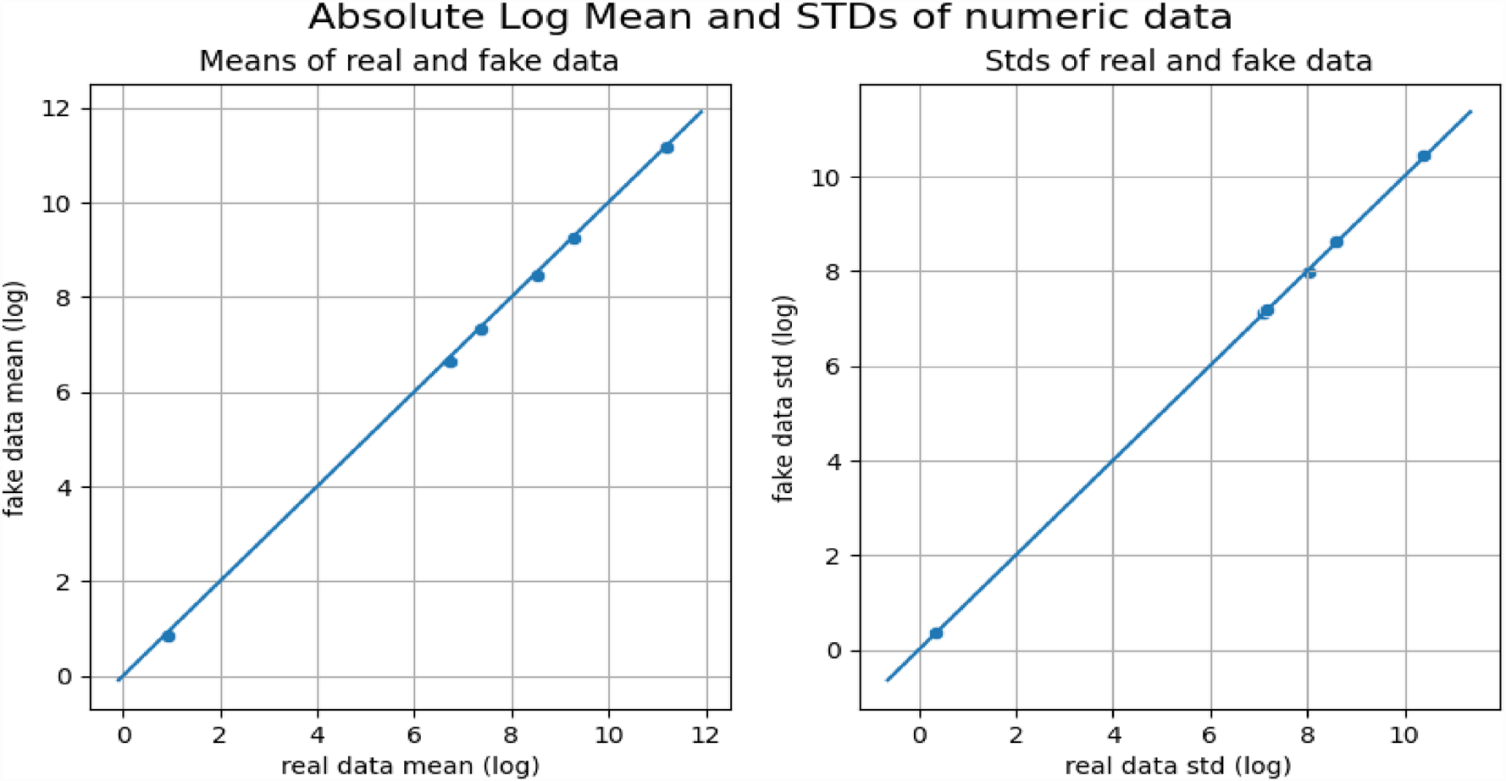
The absolute log means and std between the R and FGC datasets.

**Figure 7:**
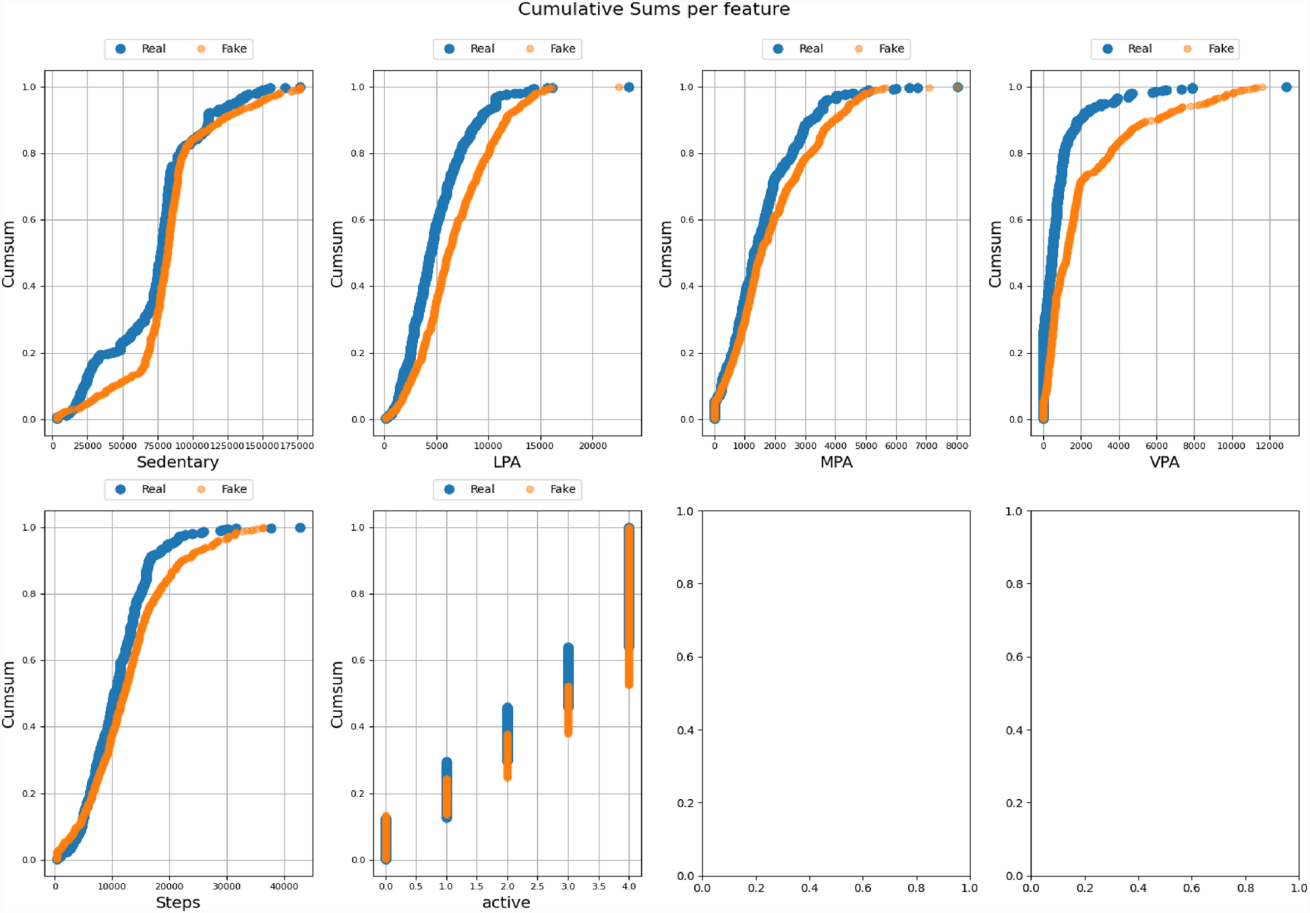
The cumulative sums per feature between the R and FC datasets.

**Figure 8:**
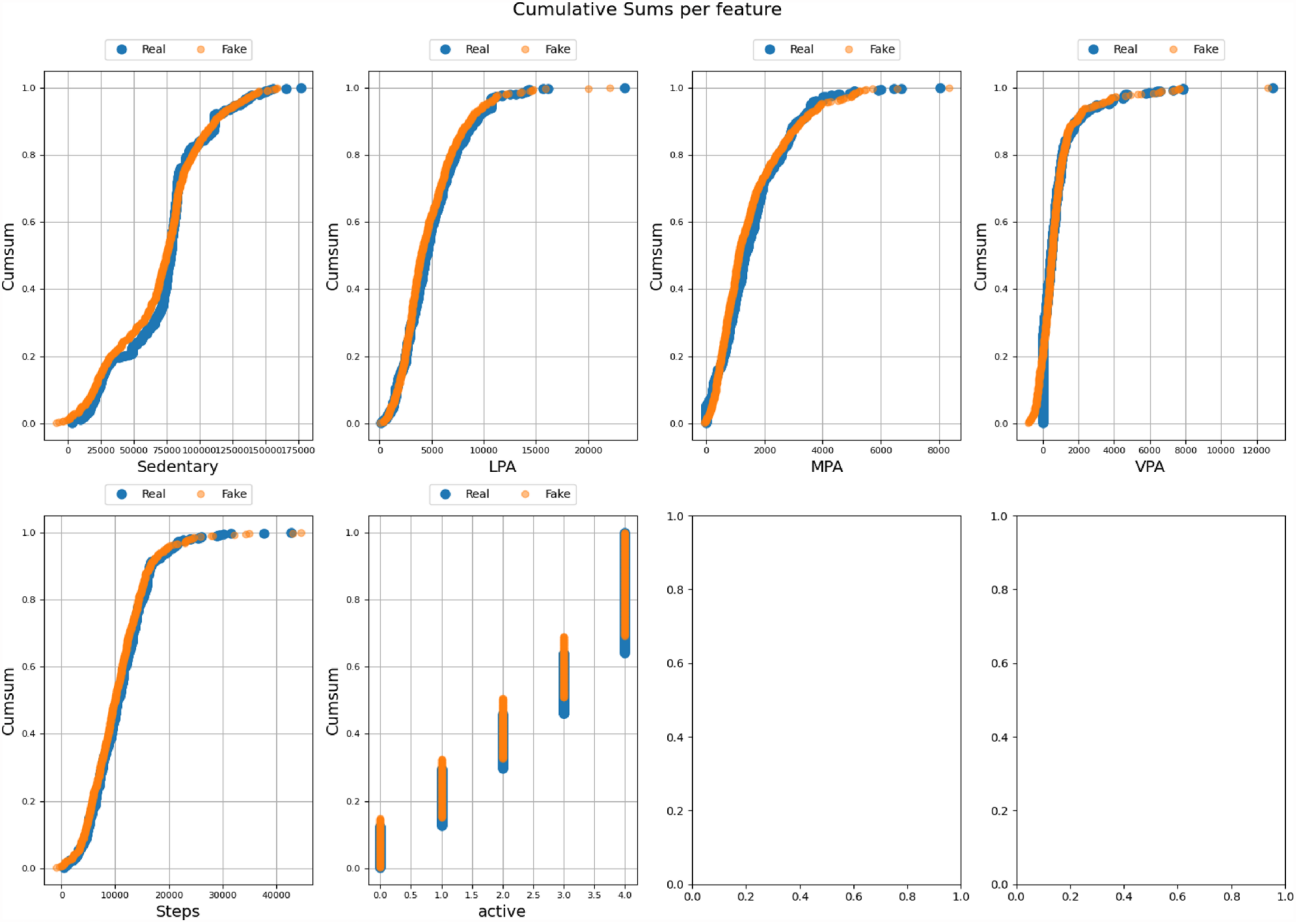
The cumulative sums per feature between the R and FGC datasets.

**Figure 9:**
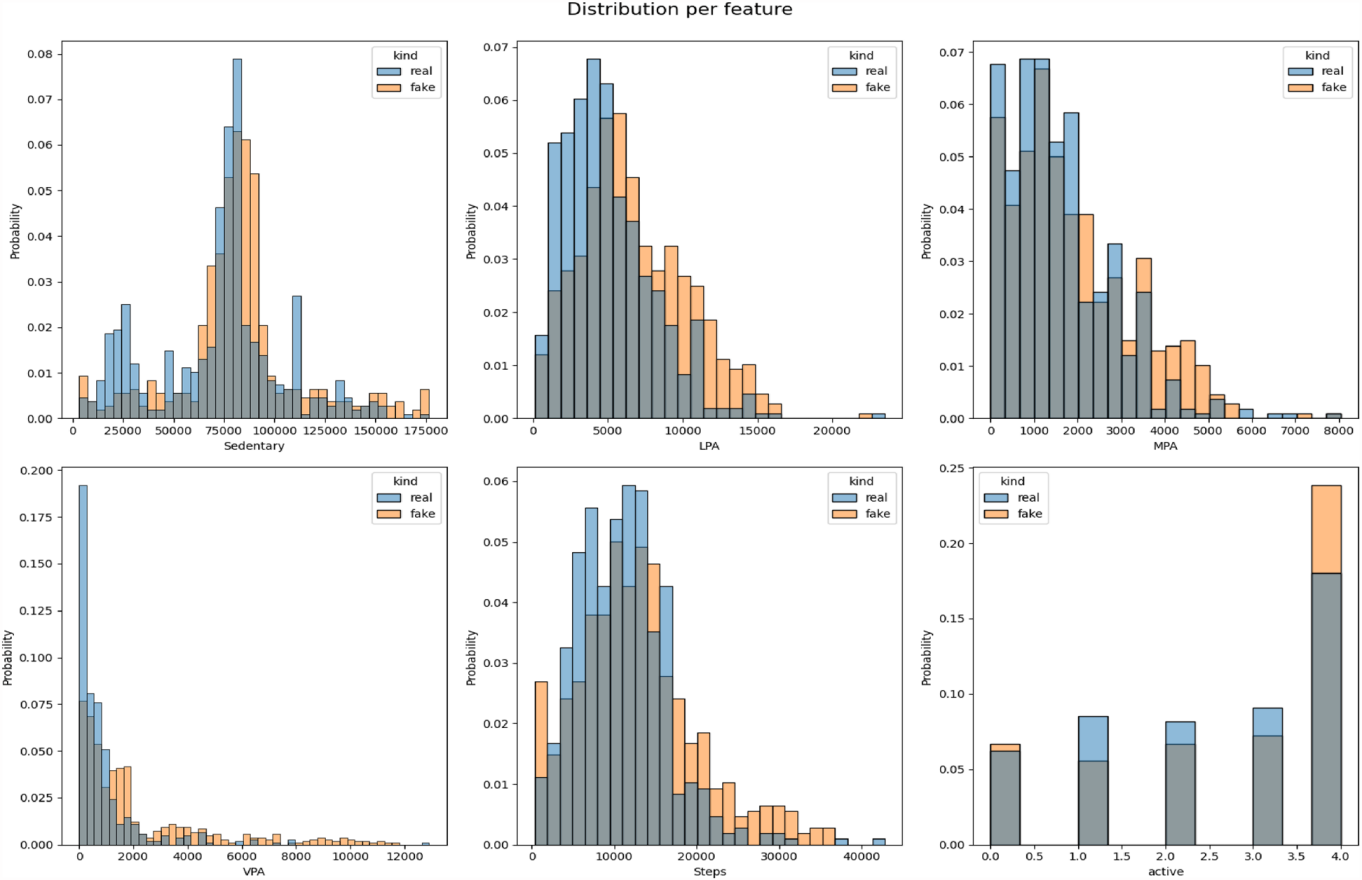
The distribution per feature between the R and FC datasets.

**Figure 10:**
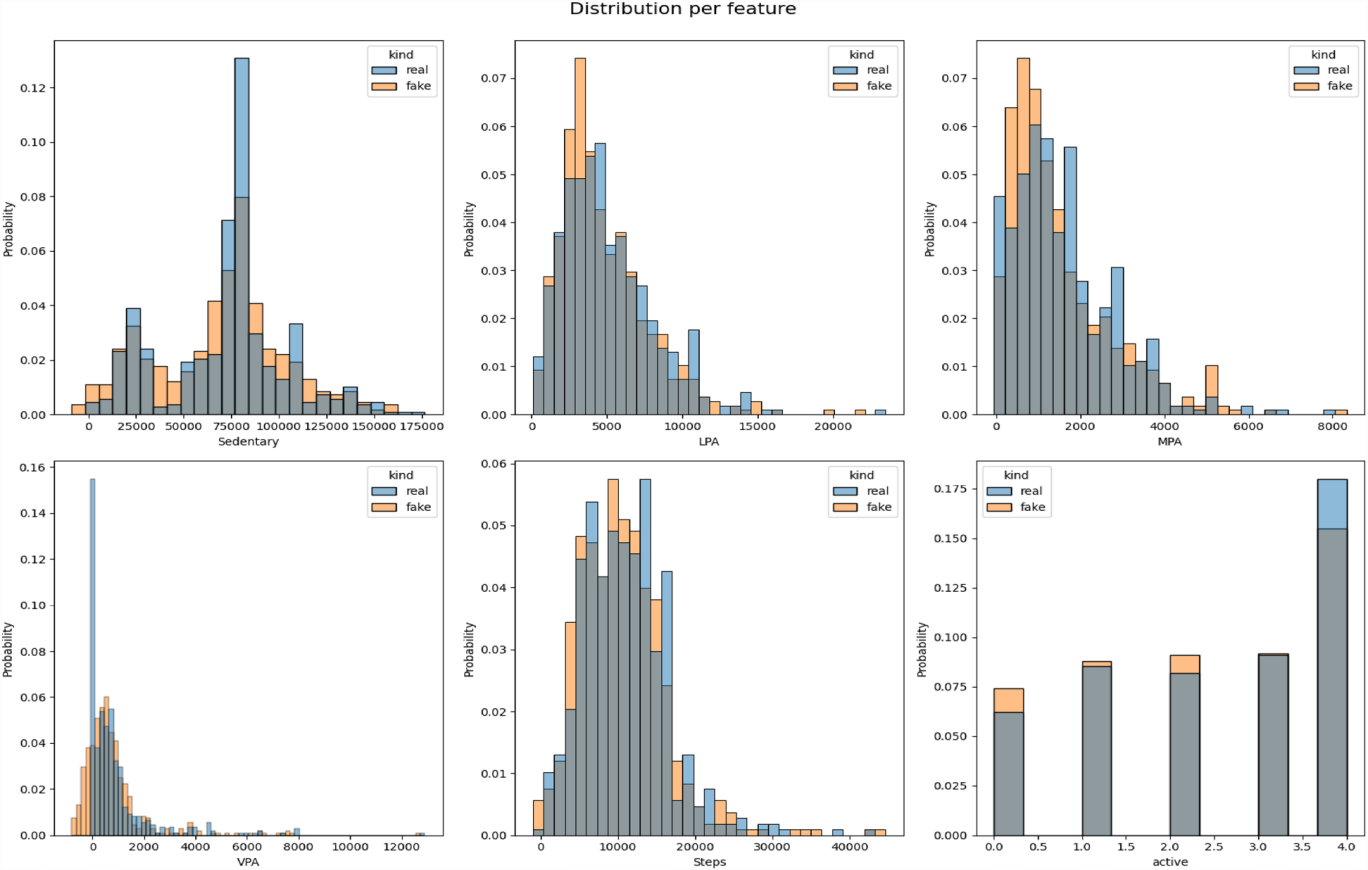
The distribution per feature between the R and FC datasets.

All the datasets have been compared with real datasets against Ordinary Least Squares or OLS (see Fig. 4), Jaccard Similarity score (see Supplementary Material-5), absolute log means and standard deviations (Figs. 5 and 6), cumulative sums per feature (Figs. 7 and 8), and distribution per feature (Figs. 9 and 10) between different datasets. We found no evidence of more than one class in FT datasets; therefore, the Jaccard Similarity score has not been compatible. OLS charts play a key role in linear regression analysis by providing visual insights into model fit, residuals, outliers, and compliance with model assumptions.

Metrics such as F1 score and Jaccard similarity score can be used for classification and measuring similarity, but they serve different purposes. The F1 score evaluates the performance of the classifier, and the Jaccard similarity score quantifies the similarity between sentences. Although they can be used together to evaluate classifier performance and the similarity between predicted and actual label sets, they are not directly interchangeable.

Statistical measures such as cumulative sums per feature, distribution per feature, absolute log means and std between two datasets serve different purposes when comparing two data sets. Cumulative totals help to track data trends, distributions reveal data characteristics, absolute logs quantify average feature differences, and standard deviations highlight changes in data distributions. Together they provide valuable insights for data comparison, anomaly detection, and decision-making in a variety of analytical environments.

### Experimental setup

We used Python 3.9.15 libraries, such as pandas (v. 1.5.2), NumPy (v. 1.22.4), SciPy (v. 1.7.3), Matplotlib (v. 3.6.2), Seaborn (v. 0.12.0), Plotly (v. 5.11.0), Keras (v. 2.10.0), Statsmodels (v. 0.13.2), SDV (v. 0.17.1), and Graph Viz (v. 0.20.1) to process data and build the AI models. We have set up the Python environment in the Windows 10 operating system using Anaconda distribution and used the Jupiter Notebook v. 6.5.2 for the development, model analysis, and data visualization. The targeted system consists of 16 GB RAM and 64-bit architecture. As the dataset is small, we performed the overall experiment on Central Processing Unit (CPU). Moreover, we used complementary open-source tools, such as Protege (v. 5.x) and Apache Jena for the design, development and management of semantic data and ontologies.

### Ontology evaluation and querying

Protege is typically used for the visual development and management of ontology, making it easier for ontology engineers and domain experts to create and edit. Once ontology was created, we used Apache Jena applications for semantic data processing and reasoning. Apache Jena leveraged for data integration, querying, and reasoning with RDF data management, ontology reasoning, SPARQL query, and integration. Jena helped in In-Memory ontology persistence with triple store database (TDB). The Fuseki server and ARQ engine helped in remote federated querying and REST-style interaction during SPARQL query processing. The querying of subject, predicates in the ontology and the loading of ontology took approximated < 0.3 s. We used the Hermit reasoner from Protégé editor (V.5.x) for checking the structural consistency of the proposed ontology model as it performed the best (execution time ≈ 1 s) as compared to other reasoners, such as Pellet, RacerPro, Fact++. Supplementary Material-6 represents sample SPARQL queries that demonstrate we retrieved essential information from the proposed ontology as presented in Supplementary Material-2. Our OWL ontology supports OWL Full specification which is a variant of OWL with its own set of logic and reasoning characteristics. The OWL Document Manager facilitated the creation and management of OWL ontology, to leverage the expressiveness of predicate logic within OWL.

### Evaluation outcomes

**Figure 11:**
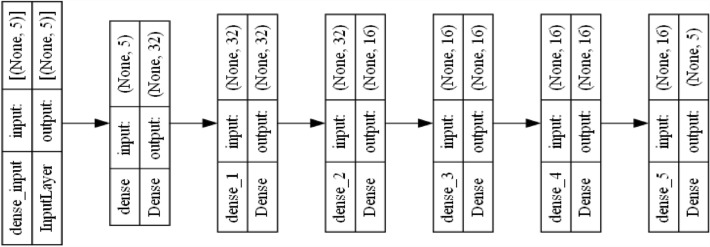
The structure of the designed and developed MLP model for classification with 2405 trainable parameters.

According to the result, the FT data produced biased values after 50,000 epochs. Therefore, we excluded the FT dataset to determine MLP classification model’s (see Fig. 11) performance in comparison with the real dataset (see Tables 7, 8, 9 and 10). To determine the model classification efficiency, we trained a MLP classifier using the following data samples—R, FGC, FC, FGC + R, FC + R and GC + FC + R. In FGC + R, FC + R and GC + FC + R, we trained the MLP model with synthetic data and perform classification on the real data, following the transfer learning approach. The average classification outcomes of four executions have been captured in Tables 7, 8, 9 and 10. In our pervious study^9^, we compared the predictive performances of our designed and developed MLP model with other state-of-the-art timeseries classification models, such as Rocket, MiniRocket, and MiniRocketVoting and our MLP model outperformed other classifiers on real. Furthermore, we have extended the study with a comparative predictive analysis on synthetic datasets. Therefore, in Tables 10, 11, 12 and 13, we have captured the results of these classifiers on different datasets to compare the performances. According to the results in in Tables 10, 11, 12 and 13, the synthetic datasets consistently lead to accuracy improvements.

**Table 10:** MLP classification results on different subsets.

Datasets	Records	Accuracy (%)	F1-score (%)	Precision (%)	Recall (%)	MCC (%)
R	539	71.0	72.5	74.0	71.0	69.0
FGC	539	68.0	68.0	68.0	68.0	65.0
FC	539	73.3	73.0	73.0	73.0	67.0
FGC + R	1078	77.6	77.0	77.0	78.0	71.0
FC + R	1078	81.0	81.0	80.0	81.0	76.0
FGC + FC + R	1617	87.0	87.0	86.0	87.0	83.0

**Table 11:** Rocket classification results on different subsets.

Datasets	Records	Accuracy (%)	F1-score (%)	Precision (%)	Recall (%)	MCC (%)
R	539	48.0	48.0	56.0	42.0	45.0
FGC	539	50.0	50.0	50.0	50.0	48.0
FC	539	51.0	51.0	51.0	51.0	50.0
FGC + R	1078	54.6	54.0	54.0	54.0	52.0
FC + R	1078	59.0	59.0	59.0	59.0	57.0
FGC + FC + R	1617	64.0	64.0	64.0	64.0	62.0

**Table 12:** MiniRocket classification results on different subsets.

Datasets	Records	Accuracy (%)	F1-score (%)	Precision (%)	Recall (%)	MCC (%)
R	539	51.0	50.2	58.0	45.0	49.0
FGC	539	55.0	55.0	55.0	55.0	52.0
FC	539	58.3	58.0	58.0	58.0	54.0
FGC + R	1078	61.0	61.0	61.0	61.0	59.0
FC + R	1078	65.0	65.0	65.0	65.0	63.0
FGC + FC + R	1617	70.0	70.0	70.0	70.0	68.0

**Table 13:** MiniRocketVoting classification results on different subsets.

Datasets	Records	Accuracy (%)	F1-score (%)	Precision (%)	Recall (%)	MCC (%)
R	539	42.0	41.3	44.0	39.0	41.0
FGC	539	44.0	44.0	44.0	44.0	43.0
FC	539	47.0	47.0	47.0	47.0	44.0
FGC + R	1078	50.0	50.0	50.0	50.0	48.0
FC + R	1078	54.0	54.0	54.0	54.0	52.0
FGC + FC + R	1617	59.0	59.0	59.0	59.0	57.0

The Rocket, MiniRocket, and MiniRocketVoting classifiers are all part of the "Rocket" family of algorithms, which are designed for efficient and effective timeseries classification. These algorithms were introduced to address challenges in processing timeseries data, such as high dimensionality and the need for computationally efficient feature extraction. While these classifiers offer advantages, it's essential to note that their performance may vary based on the specific characteristics of the dataset and the requirements of the classification task.

The importance of traditional MLP models compared to other state-of-the-art classifiers depends on the specific problem, data set size, data set type, and available resources. Careful model selection and hyperparameter tuning are crucial to realize their full potential.

## Discussion

This section discusses the outcome of the technical validation, the advantages of synthetic data generation in healthcare using well-established methods, such as GC, CTGAN, and TABGAN, and challenges associated with our data collection.

### Principal findings

The proposed ontology with integrated SSN representation enables more detailed modeling and querying of physical activity observations, including activity level, number of steps, sensors, and observation time. The proposed ontology is a simplified structure that does not only support data integration, semantic understanding, sensory observation in a structured way, but also supports standardization, interoperability, semantic modeling of predictive analysis based on sensory observations, proper reasoning, and easy querying for knowledge retrieval.

Based on the experimental evaluation, Jaccard Similarity reveals that GC produced better synthetic data samples than the CTGAN method with a close cumulative sum per feature. According to the OSL statistics FGC and FC datasets are close to the real MOX2-5 d2ataset based on individual and cumulative variable evaluation. However, GC achieved a better pairwise correlation accuracy, whereas CTGAN achieved a better accuracy (see Table 10). According to Table 10, CTGAN achieved a predictive performance that is better than what we achieved with real data and FGC data. Moreover, we have shown that the MLP model has improved its classification accuracy with increasing volume of data as it helped the MLP to understand the data pattern better. The TABGAN method has not been fruitful for this MOX2-5 datasets. In the future, we can extend this study for scalability analysis of deep learning models and other eHealth applications (e.g., eCoaching).

Modern smartphones are equipped with a variety of sensors, such as accelerometers, gyroscopes, and even barometers. These sensors are used to provide more advanced step counting functionality compared to traditional pedometers. While pedometers rely on relatively simple mechanisms and thresholds to count steps, smartphones, smartwatches, and smartphone apps use a combination of advanced sensors and complex algorithms to provide more accurate and versatile step counting capabilities. However, they all are not medically approved (CE-certified) like MOX2-5. As a part of calibration check we used our MOX2-5 sensor with other devices, such as modern smartphones (e.g., OnePlus 6 T, Samsung Galaxy, Nokia), smartwatches (e.g., Samsung Galaxy), and smartphone apps (such as pedometer, Racer, Pacer). We asked six individuals (Male: 4, Female: 2) to record step count over 2 km (km) for a duration of seven days. We found that MOX2-5 recorded 75–100 steps more on average. It seems that the accelerometry algorithm used in MOX2-5 is very sensitive in detecting thresholds.

### Importance of synthetic data generation in healthcare

We have shown a direction to use GC, CTGAN, and TABGAN on top of the real MOX2-5 datasets to do a comparative analysis and show that MLP model efficiency grows with the increasing volume of training data. The synthetic data generation process will be helpful in the creation of a robust method for the classification of activity types. The use of synthetic data may open opportunities for large-scale data sharing, model scalability, model efficiency, quality control, diversity, experimentation, availability, and analysis without revealing sensitive information.

Though the used real MOX2-5 dataset is small, we have shown a direction to use the best data synthetization method to use on real datasets for generating synthetic data in a large scale. It can be helpful for other research communities based on their research focus and needs. We would like to emphasize that such a synthetic dataset can provide unique benefits that may not be achievable with real data alone. The use of synthetic dataset may have the following advantages.

- Privacy: Synthetic data can help to address privacy concerns and protect sensitive information. In many cases, it may be difficult or impossible to access or share real data due to privacy regulations or ethical considerations. By using synthetic data, researchers can create realistic and representative datasets without compromising privacy.
- Adding more data points: The synthetic data can be used to augment existing datasets, providing more data points and a wider range of scenarios to test hypotheses. This can help to increase statistical power and improve the robustness of analyses.
- Cost Effectiveness: The synthetic data can be used to simulate scenarios that are not currently feasible to observe in real life. For example, it may be difficult or costly to collect data on rare diseases or events, or to study the effects of interventions that cannot be ethically or practically tested on human subjects. Synthetic data can be used to simulate these scenarios and generate valuable insights. Generating synthetic data can be less expensive than collecting and processing real data. This is especially useful in situations where the cost of obtaining real-world data is prohibitive, such in large-scale simulation or experimental studies.
- Diversity: Synthetic data can be used to create a wide variety of scenarios and conditions that may not be observed in real-world data. This is useful when the goal is to test the robustness of a model or algorithm under different conditions.
- Quality Control: Synthetic data can be used to create high-quality datasets with well-known ground-truth labels. This is useful for benchmarking algorithms and evaluating their performance in a controlled environment.
- Availability: In some cases, real data may not be available due to legal, ethical, or practical constraints. In these cases, synthetic data can be used as surrogate indicators so that researchers and practitioners can still make progress toward their goals.

### Challenges associated with data collection

Recruiting participants for sensor-based activity data collection in Norway, like in any research involving human subjects, comes with its own set of challenges, such as—a. *Privacy Concerns*: Norway has strict data protection laws such as the GDPR, which requires researchers to obtain informed consent and ensure the privacy and security of participant data. Solving these problems can be time-consuming and complex, b. *Informed Consent*: Obtaining informed consent from participants is critical, but explaining the technical aspects of sensor data collection to non-technical participants can be difficult. It is important to ensure that participants understand what data is being collected and how it will be used, c. *Recruitment Channels*: Identifying appropriate recruitment channels to reach potential participants can be challenging. It involves working with healthcare facilities and community organizations and online platforms to find suitable candidates, d. *Sample Representativeness*: It is difficult to ensure that the sample of participants is representative of the broader population. Bias may occur if certain groups are more willing or able to participate in sensor-based data collection studies, e. *Technology Literacy*: The success of sensor-based data collection depends on the ability of participants to interact with and understand the technology involved. Ensuring that participants have the necessary technological literacy can be challenging, especially for older or less tech-savvy populations, f. *Participant Compliance*: Participants must follow instructions to always wear or use the sensor. Maintaining participant compliance throughout the study can be challenging as some may forget to use the devices or feel uncomfortable, g. *Data Quality*: Ensuring the quality of the data collected is critical. Technical issues, sensor failure, or incorrect use by participants may cause data inaccuracies, h. *Ethical Considerations*: Researchers must consider the ethical implications of sensor-based data collection, especially when the data collected may reveal sensitive information about participants, i. *Recruitment Costs*: From purchasing and maintaining sensors to participant incentives, sensor-based data collection research can be costly. Securing adequate funding can be challenging, and j. *Cultural and Social Factors*: Norway has a diverse population, and various cultural and social factors may influence participants' willingness to study. It is important to pay attention to these factors and adjust your recruitment strategy accordingly.

To address these challenges, it is important to work with local research ethics committees to ensure transparent communication with potential participants and to adopt strategies to make participation as accessible and engaging as possible.

## Conclusions

In this work, we present the MOX2-5 dataset, its synthetic version, and some baseline experiments. We elaborated the semantification rule for annotating sensory observation in SSN Ontology for knowledge representation, semantic search, data integration, reasoning, and querying. The choice between SSN and a general ontology hinge on the particular use case and needs. General ontologies, such as RDF or OWL excel in broader knowledge representation and may be better suited for applications beyond sensor data and IoT domains. Moreover, we explained the real physical activity data collection process with the MOX2-5 activity sensor from sixteen real participants and associated challenges. Secondly, we used different synthetic data generation methods, such as GC, CTGAN, and TABGAN for generating synthetic subsets of the real data (FGC, FC, and FT) as the data volume had been small. We then compared the real data (R) with the generated data (FGC, FC, and FT) for individual and cumulative features. We then used all the real data and the subsets (R, FGC, FC, FGC + R, FC + R, GC + FC + R. In FGC + R, FC + R, GC + FC + R) for predictive analysis with our designed and developed MLP model. We found that the TABGAN method is not suitable for this real MOX2-5 dataset, GC and CTGAN methods are neck-to-neck; however, the FC dataset produced better accuracy than the other subsets. All the real and synthetic subsets of the dataset and corresponding experiments are publicly available for study replication and future studies.

### Supplementary Information

Supplementary Information 1.

Supplementary Information 2.

Supplementary Information 3.

Supplementary Information 4.

Supplementary Information 5.

Supplementary Information 6.

## Data Availability

All data generated or analyzed during this study in progress are available in the public GitHub repository. Moreover, datasets are available with this paper as supplementary files in CSV format. AC can be contacted if someone wants to have more clarification. GitHub: https://github.com/ayan1c2/ActivityClassification.git.

## Abbreviations

StaRI: Standards for reporting implementation
DL: Deep learning
MLP: Multi-layer perceptron
GC: Gaussian Capula
CTGAN: Conditional Tabular General Adversarial Network
TBGAN: Tabular General Adversarial Network
OLS: Ordinary least squares
IMA: Physical activity intensity
LPA: Low physical activity
MPA: Medium physical activity
VPA: Vigorous physical activity
GDPR: General data protection regulation
SPARQL: SPARQL Protocol and RDF Query Language
UiA: University of Agder
HOST: Holistic systems
