# Supplementary material for: Semantic representation and comparative analysis of physical activity sensor observations using MOX2-5 sensor in real and synthetic datasets: a proof-of-concept-study
Source: Sci Rep. 2024 Feb 26;14:4634. doi: 10.1038/s41598-024-55183-6 (PMC10897381; doi:10.1038/s41598-024-55183-6)
Supplement: Supplementary file 2 — Supplementary Information 2. [file 41598_2024_55183_MOESM2_ESM.pdf]

Supplementary Material as OWL Representation of the Proposed Ontology in the Turtle Format

```
#Prefixes
@prefix ex: <http://example.org#> .
@prefix ssn: <http://www.w3.org/ns/ssn/> .
@prefix rdf: <http://www.w3.org/1999/02/22-rdf-syntax-ns#> .
@prefix rdfs: <http://www.w3.org/2000/01/rdf-schema#> .

# Classes
ex:PhysicalActivityObservation rdf:type ssn:Observation ;
ex:observedActivityLevel ex:LightActivity ;
ex:observedSteps ex:StepsObservation ;
ex:observedSedentaryTime ex:SedentaryTimeObservation ;
ex:observedBySensor ex:Sensor123 ;
ex:observationTime "2023-09-16T10:00:00"^^xsd:dateTime ;
ex:hasPerson ex:AC .

ex:LightActivity rdf:type ex:ActivityLevel .

# Properties
ex:observedActivityLevel rdf:type owl:ObjectProperty .
ex:observedSteps rdf:type owl:ObjectProperty .
ex:observedSedentaryTime rdf:type owl:ObjectProperty .
ex:observedBySensor rdf:type owl:ObjectProperty .
ex:observationTime rdf:type owl:DatatypeProperty .
ex:predictedActivityLevel rdf:type owl:DatatypeProperty .
ex:hasPerson rdf:type owl:ObjectProperty .
```
