# Supplementary material for: Semantic representation and comparative analysis of physical activity sensor observations using MOX2-5 sensor in real and synthetic datasets: a proof-of-concept-study
Source: Sci Rep. 2024 Feb 26;14:4634. doi: 10.1038/s41598-024-55183-6 (PMC10897381; doi:10.1038/s41598-024-55183-6)
Supplement: Supplementary file 5 — Supplementary Information 5. [file 41598_2024_55183_MOESM5_ESM.pdf]

```
Classifier F1-scores and their Jaccard similarities::
                             f1_real  f1_fake  jaccard_similarity
index
DecisionTreeClassifier_fake   1.0000   1.0000              1.0000
DecisionTreeClassifier_real   1.0000   0.9815              0.9636
LogisticRegression_fake       0.5741   0.6759              0.4897
LogisticRegression_real       0.5741   0.3796              0.4305
MLPClassifier_fake            0.5093   0.7130              0.3012
MLPClassifier_real            0.4722   0.3889              0.2343
RandomForestClassifier_fake   0.8704   1.0000              0.7705
RandomForestClassifier_real   1.0000   0.9815              0.9636

Privacy results:
                                          result
Duplicate rows between sets (real/fake)  (109, 0)
nearest neighbor mean                     0.8104
nearest neighbor std                      0.6428

Miscellaneous results:
                                  Result
Column Correlation Distance RMSE  0.4075
Column Correlation distance MAE   0.3196

Results:
                                                result
Basic statistics                                0.9922
Correlation column correlations                 0.4587
Mean Correlation between fake and real columns  0.9660
1 - MAPE Estimator results                      0.8402
Similarity Score                                0.8143
```

Supplementary Figure S1. The Jaccard Similarity Score between the R and FC datasets.

```
Classifier F1-scores and their Jaccard similarities::
                              f1_real  f1_fake  jaccard_similarity
index
DecisionTreeClassifier_fake    1.0000   0.9907              0.9817
DecisionTreeClassifier_real    1.0000   1.0000              1.0000
LogisticRegression_fake        0.5000   0.5093              0.6000
LogisticRegression_real        0.5741   0.4722              0.5882
MLPClassifier_fake             0.4259   0.4537              0.5319
MLPClassifier_real             0.4722   0.4259              0.3500
RandomForestClassifier_fake    0.9630   0.9907              0.9115
RandomForestClassifier_real    1.0000   0.9815              0.9636

Privacy results:
                                          result
Duplicate rows between sets (real/fake)  (109, 0)
nearest neighbor mean                     0.5824
nearest neighbor std                      0.4275

Miscellaneous results:
                                  Result
Column Correlation Distance RMSE  0.0801
Column Correlation distance MAE   0.0571

Results:
                                                result
Basic statistics                                0.9965
Correlation column correlations                 0.9317
Mean Correlation between fake and real columns  0.9890
1 - MAPE Estimator results                      0.9480
Similarity Score                                0.9663
```

Supplementary Figure S2. The Jaccard Similarity Score between the R and FGC datasets.
