# Supplementary material for: Semantic representation and comparative analysis of physical activity sensor observations using MOX2-5 sensor in real and synthetic datasets: a proof-of-concept-study
Source: Sci Rep. 2024 Feb 26;14:4634. doi: 10.1038/s41598-024-55183-6 (PMC10897381; doi:10.1038/s41598-024-55183-6)
Supplement: Supplementary file 6 — Supplementary Information 6. [file 41598_2024_55183_MOESM6_ESM.pdf]

Supplementary Material: General SPARQL Queries against the Proposed Ontology

Supplementary Query Q1. Retrieve all physical activity observations

```
SELECT ?observation
WHERE {
  ?observation rdf:type ex:PhysicalActivityObservation .
}
```

Supplementary Query Q2. Find the sensor that observed a specific observation

```
SELECT ?sensor
WHERE {
  ex:ObservationXYZ ex:observedBySensor ?sensor .
}
```

Supplementary Query Q3. List all observations with a specific activity level

```
SELECT ?observation
WHERE {
  ?observation ex:observedActivityLevel ex:LightActivity .
}
```

Supplementary Query Q4. Calculate the total number of steps observed

```
SELECT (SUM(?stepsCount) as ?totalSteps)
WHERE {
  ?observation ex:observedSteps ?stepsObservation .
  ?stepsObservation ex:observedStepsCount ?stepsCount .
}
```

Supplementary Query Q5. Find observations made during a specific time period

```
SELECT ?observation
WHERE {
```

```
  ?observation rdf:type ex:PhysicalActivityObservation .

  ?observation ex:observationTime ?time .

  FILTER (?time >= "2023-09-16T08:00:00"^^xsd:dateTime && ?time <= "2023-09-16T18:00:00"^^xsd:dateTime)

}
```

Supplementary Query Q6. Retrieve the sedentary time of a specific observation

```
SELECT ?sedentaryTime

WHERE {

  ex:ObservationXYZ ex:sedentaryTime ?sedentaryTime .

}
```

Individualized SPARQL Queries

Retrieve all physical activity observations for a specific person

```
SELECT ?observation
WHERE {
  ?observation rdf:type ex:PhysicalActivityObservation .
  ?observation ex:hasPerson ex:AC .
}
```

Calculate the total number of steps observed for a specific person

```
SELECT (SUM(?stepsCount) as ?totalSteps)
WHERE {
  ?observation ex:observedSteps ?stepsObservation .
  ?stepsObservation ex:observedStepsCount ?stepsCount .
  ?observation ex:hasPerson ex:AC .
}
```

Retrieve the predicted activity level for a specific person

```
SELECT ?predictedLevel
WHERE {
  ?observation ex:predictedActivityLevel ?predictedLevel .
  ?observation ex:hasPerson ex:JohnDoe .
}
```
